# Severe intrahepatic cholestasis of pregnancy due to a Sertoli-Leydig cell tumour in a woman with polycystic ovary syndrome: a case report

**DOI:** 10.1186/s12884-022-05159-z

**Published:** 2022-11-02

**Authors:** Feng Yun, Leyi Fu, Dong Xu, Fan Qu, Fangfang Wang

**Affiliations:** Women’s hospital, School of Medicine, Zhejiang University, 310006 Hangzhou, China

**Keywords:** Intrahepatic cholestasis of pregnancy, Polycystic ovary syndrome, Sertoli–leydig cell tumour, Hyperandrogenism, Case report

## Abstract

**Background:**

Intrahepatic cholestasis of pregnancy (ICP) is a common gestational complication characterized by pruritus and elevated bile acids, usually occurring in the third trimester when the serum estrogen and progesterone levels are highest. Hyperandrogenism during pregnancy is a pathological state that is mostly induced by polycystic ovary syndrome (PCOS) but rarely by concomitant androgen-secreting ovarian tumours. To date, no correlation has been drawn between ICP and hyperandrogenism.

**Case presentation:**

Here, we present a rare case of early-onset severe ICP in a PCOS patient conceived via in vitro fertilization-embryo transfer, with worsening hirsutism and acne due to high levels of testosterone and dehydroepiandrosterone sulphate, both of which were produced by a fast-growing ovarian Sertoli–Leydig cell tumour. Her serum estradiol was also very high, which was speculated to be converted from the circulating androgens by the placenta. She had preterm premature rupture of membranes and delivered at 30 weeks, followed by a rapid remission of ICP as her serum estradiol dropped. However, the excessive androgens did not retreat until the large ovarian tumour was surgically removed.

**Conclusion:**

This unusual case highlights the concurrence of original hyperandrogenism and subsequent hyperestrogenism during pregnancy and the resultant confounding manifestations. Obstetricians should be aware of the potential association between androgen excess and ICP via placental aromatization.

## Background

Intrahepatic cholestasis of pregnancy (ICP) is a common gestational complication characterized by pruritus and elevated bile acids, which affects 1%–4% of pregnancies in different ethnic populations [1]. Adverse foetal outcomes are associated with severe ICP defined as serum total bile acids (TBA) ≥ 40 µmol/L, involving spontaneous preterm labour, asphyxia, cardiac arrhythmias, pulmonary dysfunction, meconium staining of amniotic fluid, and even intrauterine death when TBA ≥ 100 µmol/L [2, 3]. Growing evidence from human and animal studies have implicated both estrogen and progesterone, which physiologically increase along with gestation, as the primary and direct cause of ICP via the disruption of bile acid metabolism [1].

Maternal androgens also increase to a lesser degree during normal pregnancy, and pregnancy-induced mechanisms can protect both the mother and fetus from the detrimental effects of androgen excess [4]. However, several pathological conditions may disrupt this balance by the enormous production and/or impaired clearance of androgens, leading to gestational hyperandrogenic states. Among them, polycystic ovary syndrome (PCOS) is the most common cause, affecting 5%–10% of women of reproductive age [5]. Other infrequent maternal causes include hyperreaction luteinalis, pregnancy luteoma, and concomitant ovarian or adrenal tumour in rare instances [4]. Here, we present a rare case of ICP induced by an androgen-secreting ovarian tumour in a woman with PCOS.

## Case presentation

A 28-year-old Chinese primigravida presented at 29 weeks of gestation with an enlarging ovarian mass for four months and exacerbating pruritus for more than a month. She had been diagnosed with PCOS for two years because of oligomenorrhea and hyperandrogenaemia and finally conceived a singleton pregnancy via in vitro fertilization-embryo transfer (IVF-ET). At the 12th week, a routine sonographic examination accidentally observed a 3.5*2.6 cm cyst in her right ovary. From then, she noticed that acne gradually appeared on her face, upper chest and back, which did not concern her much. By the next follow-up at the 23rd week, her right ovary had grown to 14*11 cm, with multiple anechoic cysts inside. A magnetic resonance imaging (MRI) investigation two weeks later demonstrated the multilocular, solid-cystic nature of the mass, measuring 14*17*20 cm.

In the meantime, she developed pruritus at approximately 24 weeks, which was mainly confined to the extremities and worsened at night. The serum bile acids and transaminases were all normal at first. However, when the pruritus became more severe at the 28th week, a second test revealed a markedly elevated TBA level as high as 143 µmol/L, a mild elevation of alanine aminotransferase (ALT), and abnormal tumour markers CA125 and alpha-fetoprotein (AFP) (Table 1). Primary liver and gallbladder diseases were excluded before ICP was diagnosed. Her pruritus was substantially relieved after initial treatment with 500 mg peroral ursodeoxycholic acid (UCDA) two times daily and 1500 mg intravenous S-adenosyl methionine (SAMe) once daily.

**Table 1:** Main laboratory results before delivery, after delivery and after surgery

	Reference range	2 weeks before delivery	6 days before delivery	1st day after delivery	5th day after delivery	4th day after surgery	2 weeks after surgery	6 weeks after surgery
TBA (µmol/L)	0 ~ 13	143	128	295	15	< 1	/	/
ALT (U/L)	7 ~ 40	112	96	84	72	23	/	/
E2 (pmol/L)	/	/	2.8*10^5^	2.9*10^3^	1.2*10^3^	80.2	98.4	/
P4 (nmol/L)	/	/	> 191.0	113.0	28.7	0.30	0.27	/
T (nmol/L)	0.3 ~ 3.0	/	> 52.0	> 52.0	> 52.0	0.70	< 0.1	/
DHEAS (µmol/L)	2.68 ~ 9.23	/	17.7	> 27.0	> 27.0	1.43	1.71	/
CA125 (U/mL)	0 ~ 47	321.0	422.0	672.0	788.0	521.0	249.0	21.4
AFP (ng/mL)	0 ~ 7	247.9	303.0	220.0	117.0	41.2	15.2	3.1

*TBA *Total bile acids, *ALT *Alanine transaminase, *E2 *Estradiol, *P4 *Progesterone, *T *Testosterone, *DHEAS *Dehydroepiandrosterone sulfate, *CA125 *Cancer antigen 125, *AFP *Alpha-fetoprotein

**Fig. 1:**
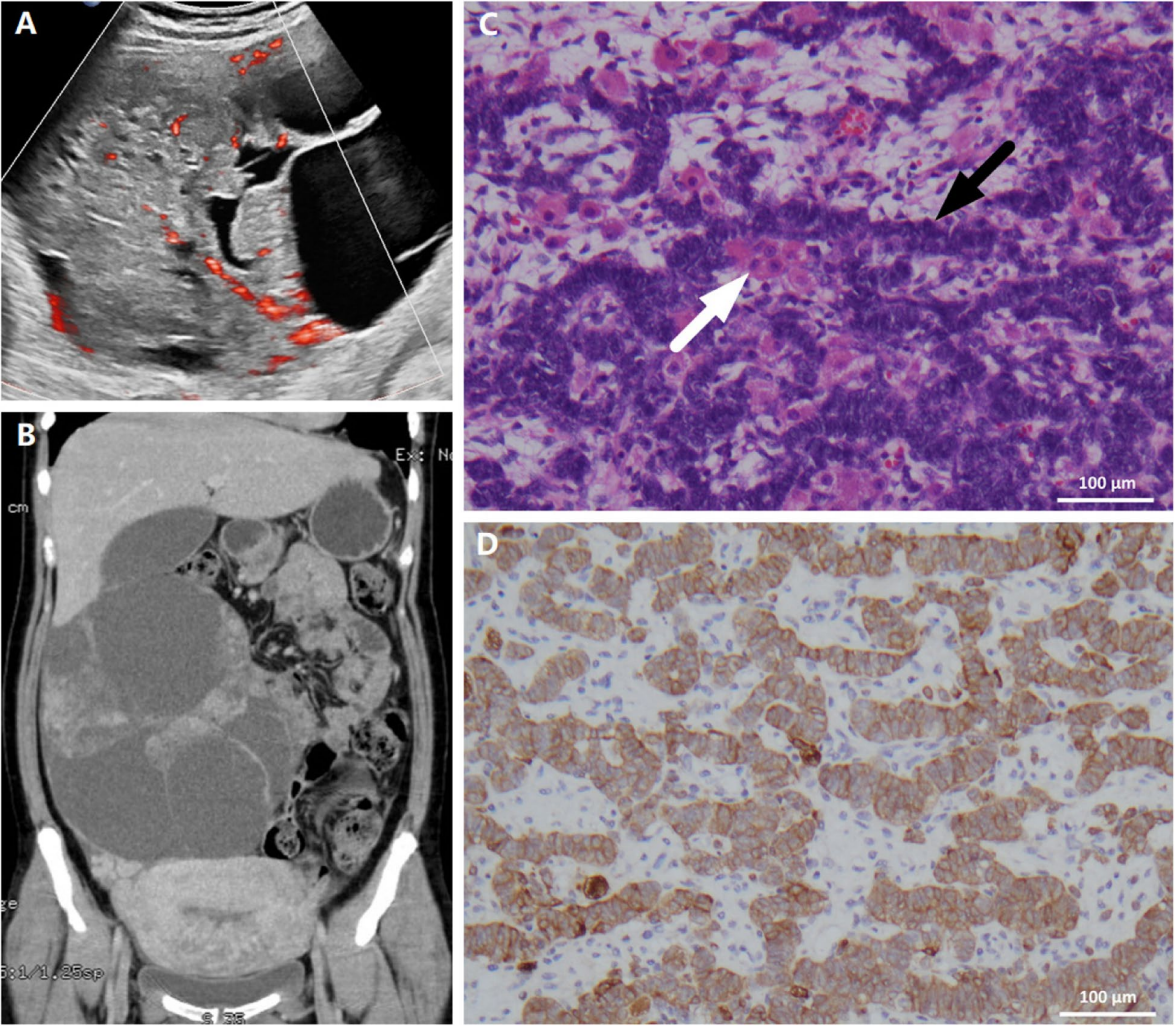
Imaging studies and pathological examination of the ovarian tumor. Microscopic images were acquired with a Leica DM2500 microscope equipped with a Nikon DS-Fi3 camera and NIS-Elements software. **A** Doppler ultrasound image before delivery showed the solid-cystic nature of the mass with medium blood perfusion. **B** Reformatted coronal contrast CT image after delivery showed the huge oval-shaped, well-demarcated, multilocular cystic mass with enhancement in the solid component occupying the right abdominal cavity. **C** Haematoxylin-eosin staining showed clusters of Sertoli cells (black arrow) which were adhered by scattered Leydig cells (white arrow) (x200). D: Immunohistochemical staining showed Sertoli cells positive for inhibin (x200)

On admission, physical examination revealed excessive hair on the lower abdominal area and the extremities with a Ferriman–Gallwey score of 16 [6] and extensive acne over her face and upper trunk, with no signs of virilization. Laboratory work-up found the following: serum testosterone > 52.0 nmol/L, dehydroepiandrosterone sulfate (DHEAS) 17.7 µmol/L, estradiol 2.8*10^5^ pmol/L, progesterone > 191.0 nmol/L. While the levels of TBA and ALT were not much reduced, both the tumour markers were progressively increasing (Table 1). Reevaluation by ultrasound showed continuous enlargement of the right ovarian cystic mass with medium blood flow (Fig. 1A), whereas the fetus appeared to be normal. However, her cervix was already 0.5 cm dilated with nearly complete effacement despite notable uterine contractions; hence, corticosteroids (4 doses of 6 mg intramuscular dexamethasone) were given in addition to UDCA and SAMe.

The patient was then reviewed by a multidisciplinary team. With full consent, she rejected immediate surgical intervention regardless of the possibility of an ovarian borderline or malignant tumour. Nevertheless, she had preterm premature rupture of membranes four days after admission, followed by the immediate onset of labour. A healthy female newborn was delivered uneventfully, weighing 1340 g at 30 weeks, with grade 3 meconium-stained amniotic fluid.

On the next day postpartum, the patient’s TBA peaked at 295 µmol/L, whereas her estradiol drastically plunged to 2.9*10^3^ pmol/L. The pruritus soon disappeared as her TBA dropped to close to normal after a couple of days. Her serum progesterone and AFP also decreased quickly, but her DHEAS and CA125 were still increasing, when the testosterone level remained above the upper limit (Table 1). Enhanced CT displayed a large oval-shaped, well-demarcated, multilocular cystic mass of 10*20*23 cm, with thickened walls and enhancement in the solid component. Also, a small number of ascites were seen, but there was no visible lymphadenopathy (Fig. 1B).

Laparotomy was performed one week after delivery. Apart from the extremely enlarged right ovary, her uterus and the left adnexa were otherwise normal, and she was negative for ascites. Thus, unilateral salpingo-oophorectomy was performed. The pathological (Fig. 1C) and immunohistochemical (Fig. 1D) examination of the tumour established the diagnosis of an intermediate differentiated Sertoli–Leydig cell tumour (SLCT), stage IC. Blood tests on the 4th postsurgical day showed regular levels of bile acids, transaminases and reproductive hormones. Her serum CA125 also decreased progressively and finally reached the normal range a month later (Table 1). The patient had full resolution after adjuvant chemotherapy with four cycles of cisplatin, etoposide, and bleomycin. Her symptoms of hirsutism and acne gradually remitted within one year and there have been no signs of recurrence so far.

## Discussion and conclusion

SLCT is an ovarian androgen-secreting tumour that belongs to the sex-cord stromal ovarian tumour group [7]. SLCT accounts for less than 0.5% of all ovarian tumours, typically affecting women of reproductive age, and up to 85% of them may present with clinical signs of hyperandrogenism, such as hirsutism and virilization [8]. The hormonal profile of SLCTs is dominated by high levels of testosterone, dehydroepiandrosterone, androstenedione, and 17-hydroxy progesterone, all of which are secreted mostly by the Leydig cell component [9, 10]. In a small proportion of patients, estrogenic manifestations are also noticeable due to high levels of serum estradiol, which is speculated to be either generated by the Sertoli cell component or converted from circulating testosterone peripherally [10, 11]. DHEAS, on the other hand, is predominantly produced by adrenal glands, and its exceptional relation to SLCTs is only reported in a couple of cases [12, 13]. During pregnancy, fetal- and maternal-originated DHEAS enters the placenta, where it is metabolized into androstenedione and testosterone, which are further catalysed into estradiol and estrone by placental aromatase enzyme (CYP19A1) [14]. This potent aromatase eliminates androgen buildup in the maternal circulation and facilitates downstream placental production of estrogens [15, 16]. In our case, both testosterone and DHEAS are secreted by SLCT and serve as the main substrates of the extremely high serum estradiol. This is indicated by the drastic reversion of maternal hyperestrogenemia, but not hyperandrogenism, after delivery. In a word, excessive androgens could result in high levels of estrogens via placental conversion during pregnancy.

ICP is attributed to genetic factors and steroid hormones [17]. Both estrogen and progesterone, as well as their metabolites, have been implicated as the etiology of ICP in human and animal studies [1]. The subsequent serum TBA elevation correlates with maternal symptoms and adverse perinatal outcomes [2, 3]. In contrast, androgens have not been found to be involved in the pathogenesis of ICP. And ICP has not been reported before in gestational hyperandrogenic conditions, among which PCOS accounts for a substantial proportion. Our case inspires the idea that androgen excess might indirectly lead to ICP. Such rarity can be partly explained by the fact that hyperandrogenism in PCOS is generally much less severe than in this case [18], so the resultant overabundance of estrogen hardly ever disrupts the bile acids metabolism in PCOS.

The diagnosis of PCOS is always made upon the exclusion of other diseases, i.e., androgen-secreting endocrinopathies and tumours [19]. The chance of PCOS concurrent with SLCT is so low that only one case has been reported in the literature [20]. In our case, the patient was diagnosed with PCOS two years before she conceived via IVF-ET, and her ovarian mass was not visible until the 1st trimester ultrasound scan. It is possible that the androgen-secreting tumour had existed since she first complained of PCOS-like symptoms but was misdiagnosed. Unfortunately, no conclusion could be reached because little is known about the natural course of SLCT, as it is often developed in an occult way [21]. On the other hand, whether pregnancy-induced hormones and IVF-ET treatment contribute to the progress of SLCT is also inconclusive. One report mentioned a fulminant SLCT during pregnancy due to a high level of LH/hCG receptor expression [22], but it seems to be rare. In general, careful tumour screening before assisted reproductive techniques is needed. Advanced imaging modalities such as CT or MRI should be applied whenever an SLCT is suspected.

Tumor markers such as CA125 and AFP have been found to be occasionally positive in SLCTs [8, 9, 13, 23]. These two nonspecific serum markers are always elevated during pregnancy and thus cannot be used in differential diagnosis. However, if they continuously grow far above the normal range set for gestation, as the serum CA125 in our case kept increasing even after delivery, it is probably an indication of a tumour. Fortunately, the majority of SLCTs are benign, with few cases having low-grade malignancy [11]. Conservative surgery is the preferred option for patients who desire fertility at an early stage, and the prognosis of SLCTs is generally good.

In conclusion, ICP has not been reported to be related to an androgen-secreting pathology in the literature. This case features concurrent hyperandrogenism and hyperestrogenism during pregnancy, which respectively lead to contradictory symptoms and have confounding laboratory results that demand clinical discernment. Obstetricians should be aware of the potential association between androgen excess and ICP via placental aromatization. And this association might be illuminated in the future by case–control studies of common hyperandrogenic conditions such as PCOS.

## Data Availability

All data generated or analysed during this study are available from the corresponding author on reasonable request.

## Abbreviations

ICP: Intrahepatic cholestasis of pregnancy
PCOS: Polycystic ovary syndrome
TBA: Total bile acids
IVF-ET: In vitro fertilization and embryo transfer
MRI: Magnetic resonance imaging
ALT: Alanine aminotransferase
CA125: Cancer antigen 125
AFP: Alpha-fetoprotein
DHEAS: Dehydroepiandrosterone sulfate
SLCT: Sertoli–Leydig cell tumour
E2: Estradiol
P4: Progesterone
T: Testosterone
LH: Luteinizing hormone
hCG: Human chorionic gonadotropin
